# The Enhanced Permeability and Retention (EPR) Effect: The Significance of the Concept and Methods to Enhance Its Application

**DOI:** 10.3390/jpm11080771

**Published:** 2021-08-06

**Authors:** Jun Wu

**Affiliations:** Center for Comparative Medicine, Beckman Research Institute of the City of Hope, 1500 East Duarte Rd, Duarte, CA 91010, USA; jwu@coh.org; Tel.: +1-626-218-3305

**Keywords:** EPR effect, nanomedicine, drug delivery, arterial infusion, canine cancer

## Abstract

Chemotherapy for human solid tumors in clinical practice is far from satisfactory. Despite the discovery and synthesis of hundreds of thousands of anticancer compounds targeting various crucial units in cancer cell proliferation and metabolism, the fundamental problem is the lack of targeting delivery of these compounds selectively into solid tumor tissue to maintain an effective concentration level for a certain length of time for drug-tumor interaction to execute anticancer activities. The enhanced permeability and retention effect (EPR effect) describes a universal pathophysiological phenomenon and mechanism in which macromolecular compounds such as albumin and other polymer-conjugated drugs beyond certain sizes (above 40 kDa) can progressively accumulate in the tumor vascularized area and thus achieve targeting delivery and retention of anticancer compounds into solid tumor tissue. Targeting therapy via the EPR effect in clinical practice is not always successful since the strength of the EPR effect varies depending on the type and location of tumors, status of blood perfusion in tumors, and the physical-chemical properties of macromolecular anticancer agents. This review highlights the significance of the concept and mechanism of the EPR effect and discusses methods for better utilizing the EPR effect in developing smarter macromolecular nanomedicine to achieve a satisfactory outcome in clinical applications.

## 1. Introduction

It is crucial to understand the pathophysiological characteristics of solid tumor growth, especially the compound transportation regulation of the tumor vasculature, in order to achieve selective drug delivery and therapeutic effects for cancer chemotherapy. It has been well observed that tumor vessels are highly permeable to macromolecular compounds. After entering tumor tissue, these macromolecular compounds are trapped inside the tumor tissue for a prolonged period of time. In 1986, Hiroshi Maeda and his colleagues from Kumamoto University School of Medicine coined the term enhanced permeability and retention effect (the EPR effect) to describe the unique pathophysiological phenomenon of the solid tumor vasculature [1]. Since this theory is very important for understanding tumor vessel transportation regulation, the EPR effect has been well accepted as one of the universal pathophysiological characteristics of solid tumors, and acts as a fundamental principle for designing and developing tumor-targeting delivery of anticancer drugs [2,3]. However, the development of nanomedicine has been frustrated for decades in achieving satisfactory therapeutic benefits in clinical practice. Therefore, the existence and intensity of the EPR effect in human solid tumor circumstances has been debated [4,5]. For example, it is considered that the EPR effect is more significant in experimental small animal tumor models than in human tumors. The delivery efficiency of nanoparticles into human tumor tissue is very low compared to that in animal tumor models. The extravasation mechanism for nanoparticles into tumors is not only via the gaps between endothelial cells in the tumor vasculature, but also via the transcellular pathways by vesiculo-vacuolar organelles (VVOs) [6]. Therefore, it is crucial to recognize the significance of the EPR effects, its pathophysiological mechanism, its pitfalls, and strategies for better harnessing this concept in drug development and clinical application.

## 2. The EPR Effect: The Universal Pathophysiological Phenomena in Rodents, Other Mammalian Animals and Human Solid Tumors

The EPR effect has been well observed and documented in solid tumors of rodents, rabbits, canines, and human patients [1,2,3,7,8,9,10,11,12,13]. It is based on several pathophysiological characteristics of solid tumors:

(1) Massive irregular neovascularization in tumors with structural and functional abnormalities in tumor blood vessels. To meet urgent demands for nutrient and oxygen supplies, the tumor vasculature is very dense and tortuous, with deficient basement membranes and fenestrated structures of endothelial tubes in some immature vessels. The pericytes and smooth muscle cells surrounding tumor blood vessels are either deficient or malfunctional in smooth muscle alpha actin when responding to blood pressure regulation stimuli [14,15,16]. Recent studies have found that the gaps between endothelial cells in tumor vessels are at low frequency, and the transendothelial pathways are the dominant mechanism of nanoparticle extravasation in tumors [17]. This is consistent with the previous observation that macromolecules are highly permeable in the mature veins or venules, constructed by a continuous endothelium with closed interendothelial cell junctions [18]. These structures render them highly permeable to nutrients, especially macromolecules, to be extravasated from tumor blood vessels into the interstitial space of tumor tissue.
(2) Elevated expression of inflammatory factors such as prostaglandins, bradykinin, nitric oxide, peroxynitrite, interleukin 1 beta, interleukin 2, interleukin 6, proteases, interferon gamma, VEGF and HIF−1 alpha. All these factors coordinate in solid tumor tissues and sustain the EPR effect [7,19,20,21].
(3) Lack of efficient drainage of lymphatic systems in solid tumor tissue. This deficiency results in the retention of extravasated macromolecules in tumor tissues, which provides the opportunity for passive targeting delivery of macromolecular anticancer drugs [1,7,22].

## 3. The Significance and Challenges in Concept and Application of the EPR Effect in Human Cancer Therapy

One of the arguments for the EPR effect concerns the roles of interstitial fluid pressure [23,24] and solid stress [25] in solid tumor tissue. The interstitial fluid pressure and solid stress exist due to the expansion of the tumor mass against surrounding normal tissue. Different from interstitial fluid pressure, the solid stress in tumors is considered to be residual stress that compresses blood vessels in tumor tissues, causing hypoxia and impeding drug delivery [26]. It is believed that interstitial fluid pressure and solid stress are the major obstacles preventing efficient delivery of macromolecules into tumor tissue [27]. However, we have seen tremendous evidence that macromolecules do accumulate in both rodent and human solid tumor tissues in size-dependent and time-dependent manners. Interstitial fluid pressure or solid stress hinders drug penetration into the center of tumor tissue, but it does not prevent the macromolecular agents from extravasating and accumulating in the peripheral area of tumor tissue. The EPR effect occurs primarily in the peritumoral area [28]. Interstitial fluid pressure and solid stress provide the mechanism for the retention effect because under such pressure or stress the formation of functional lymphatic vessels is prevented due to their collapse from the pressure [22]. The interstitial fluid pressure and heterogeneous blood supply are both observed in rodent and human solid tumors; therefore, these are not valid reasons for rebuttals that assume the EPR effect won’t function in human solid tumor tissue.

It is critical to understand and remember that the peripheral highly vascularized area is the most vigorously growing zone of tumors. The center of tumor tissues lacks blood flow and is necrotic or seminecrotic [28]. When suppressing the growing activities in peritumoral zones, tumors are restricted or eliminated. It is not necessary for an anticancer drug to penetrate into the center of a solid tumor to execute anticancer activities. For example, trastuzumab (Herceptin) is an antibody that is successful in treating Her2 positive breast cancer growth, and penetrates only into the vascularized area [29].

There are limited examples of successful nanomedicine treatments in cancer therapy. This is one of the biggest challenges for the application of the EPR effect in nanomedicine design and clinical practice. Some doubt the usefulness of the EPR effect in clinical practice by using the example of Doxil, a polyethylene glycol (PEG) modified-liposome formulation of doxorubicin, which does not appear to significantly improve the benefits of solid tumor treatment compared with parental free drug doxorubicin [4,5]. Pegylated liposomal doxorubicin does have a significantly longer half-life in blood circulation in patients and achieves about a 10~15-fold higher concentration in tumor tissues compared with surrounding normal tissues [30], which indicates that pegylated liposomal doxorubicin does accumulate into tumor tissue by the EPR effect. In a Phase III clinical trial, an overall response rate of 45.9% was achieved [31]. Pegylated liposomal doxorubicin exhibits better therapeutic benefit than that of free doxorubicin; however, the overall therapeutic outcome is still not satisfactory. The problem of therapeutic efficacy is probably due to compromised tumor cell killing properties by the pegylation of liposomes. An in vitro study found that pegylated liposomal doxorubicin had almost no cytotoxicity effect in the first 24 h, and only achieved about 12% cytotoxicity at 48 h in a colon cancer cell line HT29 [32]. Pegylation of liposomes significantly reduced the drug release rate, and also significantly reduced the cytotoxicity potency (25% vs. 75%) of anastrozole when compared with the free drug at the 72 h time point [33]. In another similar case, pegylation of liposomal cisplatin drastically decreased cytotoxic potency by increasing cytotoxicity IC50 from 2 µg/mL (free cisplatin) to 40 µg/mL (pegylated liposomal cisplatin) when compared to that at the 48 h time point [34]. Therefore, it is not the EPR effect that failed in clinical trials but the pegylation of liposomal chemo drugs that failed to achieve satisfactory cytotoxicity efficacy within 48-h period in clinical application.

The abnormality of tumor blood vessels obstructs the blood flow into tumor tissue. Since the tumor vascular formation is mainly attributed to the effects of vascular endothelial growth factor (VEGF), the application of VEGF antibodies antagonizes the effect of VEGF and temporarily normalizes tumor vasculature [35]. The tumor vascular normalization increased the uptake and penetration of fluorescent-labeled bovine serum albumin into tumor tissue, indicating that normalization of the tumor vasculature could increase the uptake of small particles (less than 20 nm in diameter) into tumor tissue, but hindered the uptake of larger particles above 125 nm in diameter [36]. The procedure of normalization could hamper the EPR effect because it decreases the permeability of large particles crossing the tumor vessel by reducing the gaps between endothelial cells in the tumor vessels. Many permeability factors like bradykinin, nitric oxide, prostaglandins are produced by infiltrating inflammatory cells and these factors may not be “corrected” by the anti-VEGF normalizing strategy. Thus, when attempting to normalize tumor vessels to improve the delivery of nanomedicine, the size of nanoparticles, the timing order of drug administration and vascular normalization could be critical to achieving the desired delivery results [36]. The normalization effect is therefore transient, limited, and highly heterogeneous in various tumor types or tumor locations. It should be combined with other modulation approaches such as hyperthermia, radiotherapy and sonoporation to enhance the EPR effect when applying vascular normalization [37].

## 4. Potential Solutions for Improving EPR Effect-Based Nanomedicine in Human Cancer Therapy

### 4.1. Better Design of Drug and Combination with EPR Effect Enhancing Modulators

The extent of the EPR effect varies between small animal tumors and human tumors by types and locations. To better utilize the EPR effect for human tumor therapy, the design of nanomedicine should be improved.

Size and physical-chemical properties such as surface charge and spatial configuration are crucial for a drug to achieve the EPR effect. Studies using serial molecular sizes of HPMA copolymers in solid tumor animal models indicated that the threshold of macromolecular molecules (drugs) to be retained and accumulated in tumor tissue is above 40 kDa [38]. Nanoparticles in the range of 100~200 nm are considered the optimal size for achieving the EPR effect in solid tumors while escaping the filtration traps of the liver and spleen [39]. Negative or neutral surface charges are also important for achieving excellent plasma half-lives longer than several hours in circulation in order to be accumulated in tumor tissue. Particles in worm-like shapes such as ellipsoidal, cylindrical and discoidal shapes, or filomicelles, can achieve better accumulation results within tumors [40]. Deformability and degradability are also important for a smarter drug to be released in the right environmental condition to execute a tumor-killing effect once it enters the tumor tissue [39,40]. As an example, HPMA copolymer-conjugated pirarubicin achieved very promising clinical therapeutic results in a patient with stage IV prostate cancer with extensive metastasis [41].

On the other hand, the delivery of macromolecular drugs can be enhanced by temporarily modulating the EPR effect in the targeted tumor tissues, such as applying adjuvants like nitric oxide donors to enhance the EPR effect to facilitate the drug delivery into tumor tissue. As mentioned before, many other inflammatory factors involved in the EPR effect can be utilized to modulate the intensity of this effect to facilitate drug extravasation, accumulation, and penetration into tumor tissue [42,43,44,45,46]. The EPR effect can also be markedly enhanced by photo-immunotherapy with antibody-photosensitizer conjugate pretreatment to achieve up to a 24-fold greater accumulation of nanomedicines in tumors. Such significant enhancement has been termed the super-enhanced permeability and retention effect [47].

### 4.2. Improving EPR Effect-Based Nanomedicine by Enhancing Blood Flow in Solid Tumor during Drug Administration

Blood flow in solid tumors is critical for the success of nanomedicine delivery via the EPR effect [44,46,48,49,50,51]. However, it is usually overlooked. One of the major differences between rodent solid tumors and human solid tumors is blood flow rate. Generally, the blood flow rate is about 800-fold higher in human normal organs than in mouse normal organs. For example, the normal flow rate for a mouse normal liver is about 1.8 mL/100 g/min, but it is about 1450 mL/100 g/mL in a typical human normal liver. The blood flow in mouse muscle is about 0.91 mL/100 g/mL, but it is about 750 mL/100 g/mL in a typical human muscle [52]. In 6 C3 HED lymphosarcoma in C3 H mice, the blood flow in large tumors was about 5.4 mL/100 g/min [51] while in human breast tumors, the mean blood flow was about 30~64.8 mL/100 g/min [53]. Higher blood flow means higher shear force in blood vessels and quicker wash off. The difference in blood flow rate between normal tissue and tumor tissue in rodents and humans may be important to explain why nanomedicines can accumulate better in rodent solid tumors than in human solid tumors.

The application of angiotensin II could be very efficient for drug delivery via increasing the blood flow into stagnated tumor blood vessels. Hori et al., demonstrated that when applying angiotensin II in rodent or human subjects, the blood flow in tumors was selectively increased up to 5.7-fold without increasing the blood flow in normal tissue [15]. This is because the systemic blood pressure increased but the tumor blood vessels remain relaxed due to a lack of response to angiotensin II. Such increases in blood flow in tumor tissue greatly improved the perfusion of blood into tumor tissue to achieve a higher magnitude of delivery of anticancer drugs into the solid tumor tissue [15,16]. Hori and his colleagues further demonstrated that tumor blood flow fluctuates due to circadian regulation. Tumor blood flow is increased during the night and the tumor doubling rate is also higher during the night. When they administered anticancer drugs during the night, the therapeutic efficacy was significantly improved [54]. Such brilliant discoveries have yet to be broadly recognized and applied in clinic settings by the nanomedicine drug developers and clinical oncologists.

### 4.3. Improving EPR Effect-Based Nanomedicine Therapeutic Effect by Arterial Infusion via Tumor Feeding Artery

Administration of nanomedicines via intravenous infusion could be problematic to achieve the desired amount of drug into tumor tissue because of high shear force to the endothelial wall brought by fast blood flow, as we discussed above. However, when administering nanomedicines via the tumor feeding artery, the strong blood flow brings more nanomedicine into tumor tissues if the size and stickiness of the nanomedicine are right for blood vessels in tumors. There are tremendous successful reports about using lipiodol for delivering SMANCS and other anticancer drugs [12,55,56] by infusion via the tumor feeding artery. However, very few other nanomedicines are designed for arterial infusion to solid tumors. It should be noted that nowadays imaging-guided catheter interventional therapy for solid tumors is very popular in clinics around the world. Radiologists are very skillful in performing interventional therapy via a tumor-feeding artery; however, the optimal nanomedicine fit for such arterial infusion is rarely available. Current low-molecular-weight drugs such as cisplatin for tumor arterial infusion are simply suspended in lipid vehicle solution. The drugs diffuse and wash out like they would in common intravenous infusion. They cannot achieve the retention effect in tumor tissue, which results in low therapeutic efficacy.

### 4.4. Improving EPR Effect-Based Nanomedicine Preclinical Development by Using Large Animal Tumor Models

The last but not least critical issue is the selection of better animal models for the preclinical development of nanomedicine. Most products of nanomedicine are developed in small rodent tumor models. The tumors in mice are either induced by carcinogens or created by genetic engineering such as knocking in or knocking out certain genes that are related to the tumor initiation. Transplantation of a tumor from donor to recipient is another major way to create syngeneic tumor models, or xenograft tumor models, by established cell lines or fresh tumor tissues. Patient-derived xenograft (PDX) models have featured in preclinical studies in recent years. However, there is a vast difference in size between mice and humans, and thus the drug absorption, metabolism, distribution and exclusion profiles, as well as the pharmacokinetic and pharmacodynamic properties of the drugs in tumor tissue, would be very different in mice and humans. The tumors induced by cell lines or tumor tissue might also result in extreme growth behavior, such as an extraordinarily large size or an abnormally fast-growing pattern that is rarely seen in human tumors. Although rodent solid tumors exhibit excellent results of the EPR effect, the strength of the EPR effect could be very different compared to that in solid tumors of human patients. Thus, the EPR effect of a nanomedicine candidate measured in rodent tumor models might not be correctly estimated for translating into human clinical application. Canine cancers are naturally occurring with full-spectrum heterogeneity of tumor cell populations, bona fide tumor microenvironments and spatial structures that faithfully reflect the intrinsic status of blood flow and interstitial pressures of tumors. Studies show that copper−64 liposomes exhibited excellent permeability and retention (tumor uptake levels at 24 h after injection achieved 3.68-fold higher than at 1 h after injection) in different canine cancers at various locations such as mammary glands, neck muscle, front paw and intranasal regions [13]. Using larger animals such as canine cancer models is, therefore, better for guiding the preclinical development of nanomedicine. Unfortunately, there are few publications featuring utilization of canine cancer models for nanomedicine development. Companion animals as translational models can provide more accurate measurements of therapeutic efficacy based on the EPR effect and, therefore, should be considered as major animal models for the development of nanomedicine.

## 5. Conclusions

The EPR effect is the fundamental pathophysiological phenomenon of solid tumors universally observed in solid tumors in rodents and humans, as well as in other mammalian species. It is also the guiding principle for developing nanomedicine (including polymer-conjugated macromolecular anticancer drugs) aimed at passive and progressive drug delivery and retention inside the tumor tissue to achieve selective and highly efficient anticancer outcomes. However due to the heterogeneous strength in various microenvironment situations, the EPR effect has been challenged for its existence and importance in nanomedicine design and application. As a matter of fact, the EPR effect has been observed in human tumor tissues for various macromolecular compounds. When discussing the heterogeneity of the EPR effect, it should be clear that the heterogeneity is confined to the strength of the accumulation and retention of the EPR effect that varies in different types of solid tumors under various tissue environments.

The real challenge is how to utilize the EPR effect in designing and improving the therapeutic efficacy of nanomedicine. There are several enhancing strategies to improve delivery and accumulation efficacies, such as optimal size and surface charge, smarter mechanism for drug release and administration kinetics. Due to their rigid structure property, the accumulation performance of nanoparticles in tumor tissues may not necessarily be the same as with other macromolecules such as linear polymers and biological macromolecules such as albumins. Therefore, further modification of nanoparticles with polymers to improve the affinity of the nanoparticles with tumor related endothelial cells may be necessary.

Blood flow plays a critical role in delivering nanomedicines into tumor tissues. Arterial infusion via a tumor-feeding artery, and the timing of using tumor blood flow enhancers or EPR effect modulators should be applied to nanomedicine to achieve better therapeutic effects. As the strength of the EPR effect is quite different between small animal tumor models and human tumors, the selection of big animal models is also critical for guiding the design of nanomedicine by properly estimating the efficacy of tumor-targeting delivery via the EPR effect. Companion animal tumor models such as canine cancer should be utilized to guide the development of nanomedicine.

When considering the EPR effect, retention efficacy is crucial because for a drug to execute anticancer activities it should maintain above a certain effective concentration level for a certain length of time in the tumor tissue to achieve a satisfactory result.

A better future of nanomedicine via the EPR effect is yet to come.

## Data Availability

Not applicable.
